# Spatial population genetic structure and colony dynamics in Damaraland mole-rats (*Fukomys damarensis*) from the southern Kalahari

**DOI:** 10.1186/s12862-021-01950-2

**Published:** 2021-12-08

**Authors:** Samantha Mynhardt, Lorraine Harris-Barnes, Paulette Bloomer, Nigel C. Bennett

**Affiliations:** 1 Molecular Ecology and Evolution Programme, Department of Biochemistry, Genetics and Microbiology, University of Pretoria, Pretoria, South Africa; 2 Department of Zoology and Entomology, University of Pretoria, Pretoria, South Africa; 3 Mammal Research Institute (MRI), University of Pretoria, Pretoria, South Africa

**Keywords:** Social mammal, Sex-biased dispersal, Dispersal distance, Ecological constraints, Reproductive skew, Population genetics

## Abstract

**Background:**

Non-random associations within and among groups of social animals can provide valuable insight into the function of group living and the evolution of social behaviour. Damaraland mole-rats (*Fukomys damarensis*) demonstrate extremely high levels of reproductive skew, and dispersal is considered to be male-biased in onset and frequency, although asymmetry in dispersal distance is yet to be investigated. Dispersal may be positively correlated with increasing favourable environmental conditions, such as rainfall, however, the effects of ecological constraints on dispersal and colony fission–fusion dynamics have not previously been demonstrated on a spatial scale. Here we provide the first spatial population genetic study for this species. We investigated genetic structure in a population of Damaraland mole-rats from the southern Kalahari in South Africa over 3 years, combining observational dispersal data from mark-recapture with population genetic data to evaluate (1) sex-bias in frequency and distance of dispersal in this species, and (2) the effect of rainfall on fission–fusion dynamics of colonies.

**Results:**

Our results demonstrate (1) that both males and females favour local dispersal but on rare occasions may disperse over distances greater than 400 m, (2) that males may disperse over greater distances than females, and (3) that males more frequently immigrate into established neighbouring colonies than females, who predominantly disperse by colony fission, i.e. multiple individuals “budding” from their native colony into a neighbouring territory, thereby establishing new colonies. Furthermore, our results demonstrate (4) elevated dispersal and colony fission in association with increased rainfall, supporting the hypothesis that rainfall may play a significant role in the maintenance and/or disruption of reproductive skew in Damaraland mole-rat populations.

**Conclusion:**

This study represents the first fine-scale spatial population genetic study in Damaraland mole-rats, and provides relevant insights into colony fission–fusion dynamics in a social and cooperatively breeding species.

**Supplementary Information:**

The online version contains supplementary material available at 10.1186/s12862-021-01950-2.

## Background

Investigating non-random associations within and among groups of social animals is essential for understanding the function of group living and the evolution of social behaviour. Population subdivision in social species may result in the formation of smaller groups, or “colonies”. These colonies are not necessarily defined by their geographical site, but rather by their social interactions, including affinities, antagonisms, hierarchy and kinship [1]. Philopatry in these species would refer to fidelity to natal colony rather than to natal environment, and similarly dispersal refers to movement and/or integration of individuals from one social group into another. Colonies may grow or shrink over time and space, split (fission) or merge (fusion) with other colonies.

Philopatry and dispersal have been extensively studied in social species and frequently address the question of asymmetry in dispersal patterns among sexes. Dispersal asymmetry depends on social organization; in mammals, male-biased dispersal and female philopatry is common, while reversed asymmetry involving female-biased dispersal is rare, and more typically observed in passerine birds [2, 3]. Numerous population genetics studies have addressed the consequences of female philopatry, male dispersal and matrilineal structure for genetic differentiation between groups. These include theoretical [4, 5] and empirical studies involving primates [6, 7]; bats [8, 9]; plateau pikas [10] and woodrats [11, 12]. Demographic events (births, deaths, migrations) drive the size and composition of colonies, and optimal colony sizes vary among species, populations and environments [13–15]. Colony fission may occur when colonies reach a maximum size, thus producing two or more new colonies, as reported in diverse species including social spiders [16], army ants [17], naked mole-rats [18, 19], marmots [20, 21] and primates [22, 23].

The African mole-rat family (Bathyergidae) displays a wide range of sociality and co-operative breeding strategies among species (reviewed in [24]). Damaraland mole-rats (*Fukomys damarensis*) demonstrate extremely high levels of skew in reproductive success, with less than 8% of individuals typically achieving direct reproductive success [25]. Populations of Damaraland mole-rats are traditionally thought to be structured into extended family groups, or colonies of variable sizes ranging from 2 to 40 individuals, typically comprising a single breeding female (the queen), together with one or two breeding males, and their offspring [24, 26]. Non-breeding colony members form part of the subordinate work force and contribute to the care of the young [27]. These subordinates may be reproductively suppressed, but are not sterile [28]. Breeding pairs are in most instances unrelated and are usually the founders of the colonies in which they breed [26].

The evolution of cooperative breeding in the Bathyergidae may be linked to habitat aridity and food distribution [27, 29–33]. The Damaraland mole-rat is endemic to the arid ecoregions of southern Africa [26]. The habitat is characterized by high temperatures, low and sporadic rainfall patterns and coarse sandy soils, typically including the thorn-scrub woodland savannas and grasslands [27, 34]. These arid habitats with low rainfall and hard, coarse soils elevate the energetic cost of burrowing, and coupled with the uneven distribution of food resources in the form of underground roots and swollen tubers of geophytes, may result in reduced foraging success [27, 31]. This could lead to selection for group living, cooperative foraging and communal care of offspring, resulting in the evolution of the extreme reproductive skew seen in this species.

Damaraland mole-rats may disperse between colonies, either temporarily or permanently, either above or below ground [35]. The success of dispersal in these harsh habitats, is dependent on predation risk [25, 29], availability of resources and the distances between neighbouring colonies [35, 36], and has been found to be male-biased [36, 37]. Despite the risks associated with dispersal, these animals seem to disperse regularly enough to maintain reasonable levels of outbreeding in most wild populations, probably since dispersal is one of the only means by which non-breeding individuals can gain reproductive opportunity with prospective unrelated mates [38].

Various ecological and demographic factors have been proposed as drivers of dispersal in mole-rats. First, escape of the dominant breeders’ control in an individual’s native colony, in order to exploit his/her lifetime reproductive success and mate with non-colony affiliates [39]. Secondly, an attempt at inbreeding avoidance by circumventing reproduction with related colony affiliates [38], and thirdly, favourable environmental conditions such as resource availability, rainfall and good soil quality, promoting dispersal [27]. Previous studies have suggested that the social structure and reproductive patterns in Damaraland mole-rats are heavily influenced by the environment they occur in [26, 27, 29, 35, 37, 40]. The frequency of dispersal is thought to be positively correlated with increasing favourable environmental conditions, such as rainfall [25, 27, 35, 37, 41], but field data showing how ecological constraints affect dispersal are scarce [24, 37, 42], and the effect of ecological constraints on dispersal and colony fission–fusion dynamics has not been demonstrated on a spatial scale. Torrents‐Ticó et al. [37] demonstrate that rainfall increases dispersal probability in both sexes, with males dispersing earlier and more frequently than females, however dispersal distances were not investigated. Thus, asymmetry among sexes in dispersal distance, as well as the nature of spatial population genetic structure in this species remain largely unknown.

Here we investigate spatial population genetic structure in Damaraland mole-rats, and combine genetic data with direct observational dispersal data through mark-recapture to evaluate (1) asymmetry in frequency and distance of dispersal in this species, and (2) the effect of rainfall on fission–fusion dynamics of colonies. We distinguish colony fission as multiple individuals “budding” from their native colony into a neighbouring territory, from regular dispersal, typically involving a single individual dispersing into another established colony or joining another individual to form a new colony. We studied a population of Damaraland mole-rats, comprising 74 colonies, in a 1700 m × 500 m study site in the Tswalu Nature Reserve in the southern Kalahari of South Africa, over a period of 3 years (2004–2006). Genetic methods offer a means of quantifying dispersal that avoids the spatial biases associated with observational data, and may more accurately reflect the long-term average pattern of sex-biased dispersal [e.g. 43–45]. However, differences in estimates from genetic vs. observational data may arise in part due to varying reproductive success of dispersers, since genetic methods effectively measure gene flow, i.e. dispersal of gametes, or genes. A combination of direct observational data and indirect population genetic data may thus provide the most robust estimates of sex-biased dispersal [e.g. 46, 47]. In cooperatively breeding species, characterizing the nature of sex differences in dispersal is particularly important for understanding localized patterns of kin structure and sex-specific patterns of cooperation and conflict [48–50], and more broadly, these characterizations can assist in evaluating competing hypotheses for the evolution of sex-biased dispersal.

## Results

### Dispersal

**Fig. 1:**
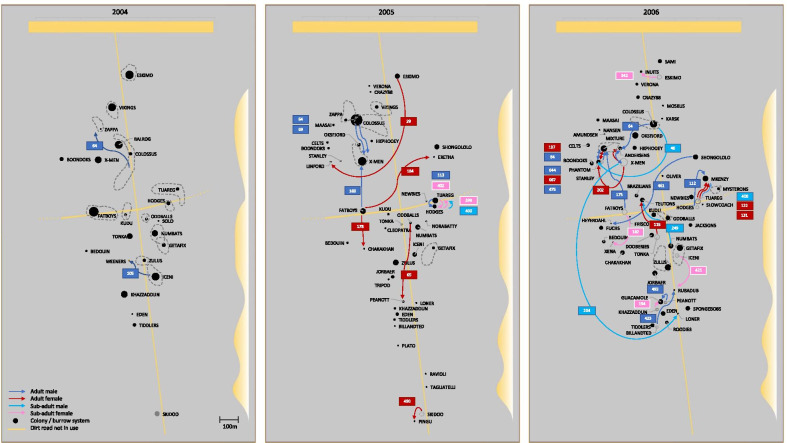
Schematic representation of the geographical distribution of colonies within the 500 m × 1700 m study site in Tswalu Kalahari Reserve. Colonies are depicted as black circles, with sizes representing sample size (ranging from 1 to 20 individuals in a given year). Colonies representing multiple sampling holes are depicted by grey dashed lines, and grey dotted circles indicate colonies not sampled (i.e. not present) in a given year. Dispersal events are indicated by coloured arrows representing adult males (dark blue), adult females (red), sub-adult males (light blue) and sub-adult females (pink), with coloured number-tags depicting the individual ID of each disperser

Mark-recapture data revealed slight geographical shifts in location and/or range of various colonies that persisted from 1 sampling year to the next (e.g. see “Colossus” in Fig. 1). Mark-recapture data also revealed movements of individuals between colonies, primarily from 1 year to the next, but in some cases within a single year. For example, sample #113 was captured at both colonies Tuareg (TUA) and Hodges (HOD) in 2005 (Fig. 1, Additional file 1: Table S1). A total of 16 male dispersers, representing 19 dispersal events (two individuals, #64 and #400, dispersed multiple times), and 17 female dispersers were recorded. Remarkably, sample #64 was captured at Colossus (COL) and Zappa (ZAP) in 2004, Colossus and X-Men (XME) in 2005 and Andersens (AND) in 2006, revealing an unusually high dispersal frequency (three dispersals in 3 years) in this particular individual. Some neighbouring colonies showed a higher frequency of dispersals than others (e.g. see Tuareg and Hodges and Mkenzy, and Mixture, Phantom and Stanley; Fig. 1). All dispersal events are illustrated in Fig. 1, in which the first panel depicts movements during the 2004 sampling period, the second depicts movements since 2004 and within the 2005 sampling period, and the third depicts movements since 2005 and within the 2006 sampling period.

**Fig. 2:**
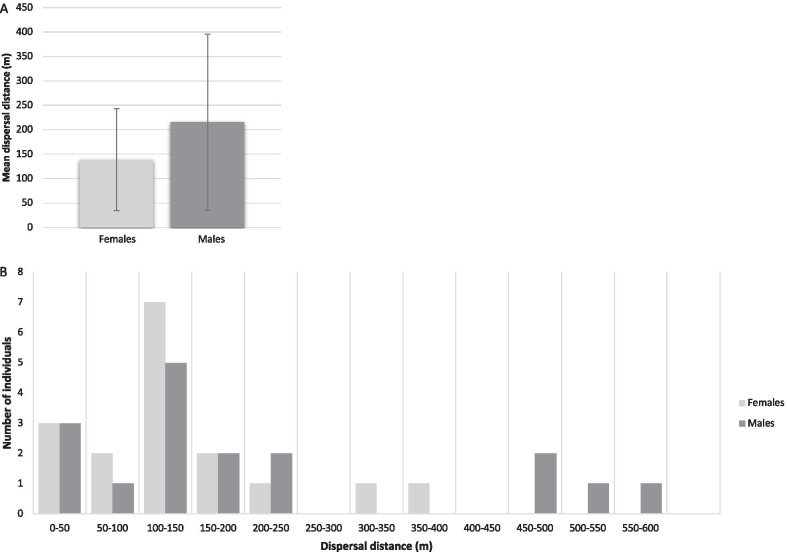
**A** Mean dispersal distance (m) for males and females, reflecting 19 male- and 16 female dispersal events. **B** Male and female dispersal distances in non-overlapping 50 m distance categories based on mark-recapture data

On average, males dispersed farther than females (mean ± SE males: 215 m ± 180 m; females: 139 m ± 104 m; Fig. 2a) although this difference is non-significant (*t*_(16)_ = 1.7; *p* = 0.11). Of the 36 (19 male; 17 female) dispersal events, only three represented distances greater than 400 m (#64: 456 m; #461: 472 m; #204: 530 m; Fig. 1, Additional file 1: Table S1), and all of these were males. Female dispersals were typically associated with establishment of new colonies (Fig. 1, Additional file 1: Table S1), with only two cases of dispersal into neighbouring colonies (12% of female dispersal events), both of which were represented by very short distances (#399 and #402; Fig. 1, Additional file 1: Table S1). Males were found to disperse more frequently into established colonies, with six such cases recorded (32% of male dispersal events).

### Population-level *F*_ST_ analysis

Across all 3 sampling years, mean *F*_ST_ for the 47 colonies, calculated using 399 individuals, was high (0.167; Table 2), indicating substantial genetic differentiation among colonies, which may be expected in cooperatively breeding species, where individuals within any given colony are on average more related to one another than they are to other individuals in the population. Females showed a higher mean *F*_ST_ than males (females: 0.175; males: 0.153; Table 2), indicating significantly greater genetic differentiation among females than males (*p* < 0.001; Table 2), a pattern consistent with females being the more philopatric sex, and males being more dispersive.

**Fig. 3:**
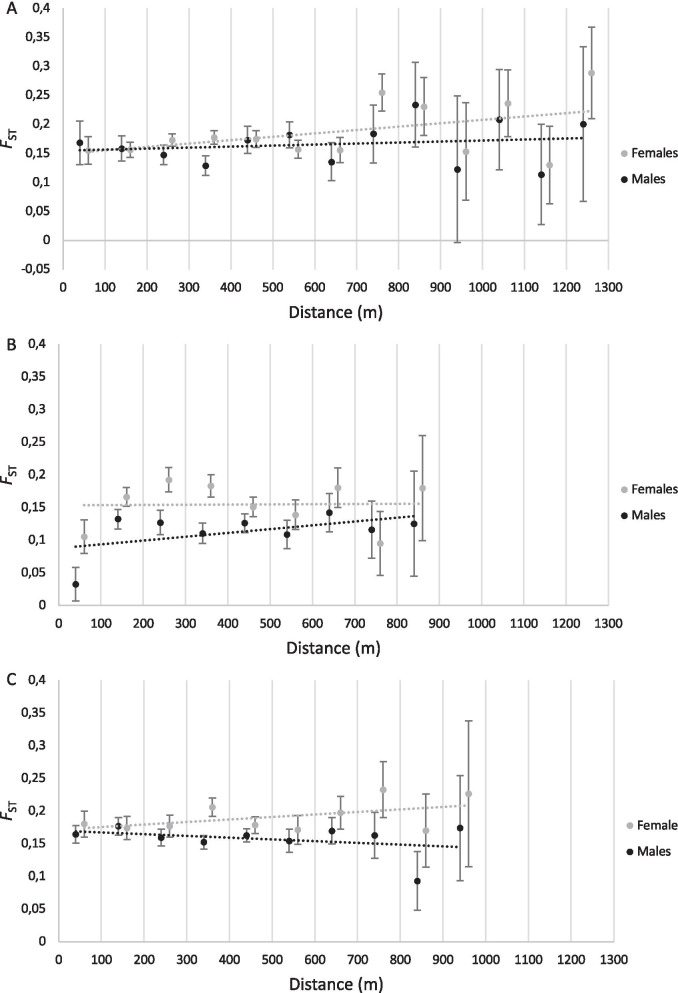
Population-level *F*_ST_ for the 47 colonies analysed in this study, including 23 colonies in 2004 (**A**), 27 colonies in 2005 (**B**), and 34 colonies in 2006 (**C**). A total of 13 distance classes of 100 m increments were specified since the maximum distance between any two colonies in our study site was 1227 m. Points represent mean *F*_ST_ for each distance class, for males and females, and error bars represent 95% confidence intervals. Dotted lines represent linear trendlines for males (black) and females (grey)

When the three datasets (2004, 2005 and 2006) were analysed separately, *F*_ST_ was found to increase with increasing spatial distance in both sexes in 2004, however, over short distances *F*_ST_ among males was higher than females, and over increasing distances *F*_ST_ became higher among females than males (Fig. 3A). This trend resulted in a non-significant overall difference in *F*_ST_ between males and females (*p* = 0.108; Table 2). In 2005, *F*_ST_ increased with increasing spatial distance in males, but remained relatively constant (on average) in females (Fig. 3B). *F*_ST_ was higher among females than males across all distance classes (except in the 700–800 m distance class). Overall, in 2005, *F*_ST_ was significantly higher among females than males (*p* < 0.001; Table 2). In 2006, *F*_ST_ increased with increasing spatial distance in females, but decreased in males (Fig. 3C). *F*_ST_ was higher among females than males across all distance classes (except in the 100–200 m distance class, where they did not differ significantly). Overall, in 2006, *F*_ST_ was significantly higher among females than males (*p* < 0.001; Table 2).

### Spatial autocorrelation of individual relatedness

**Fig. 4:**
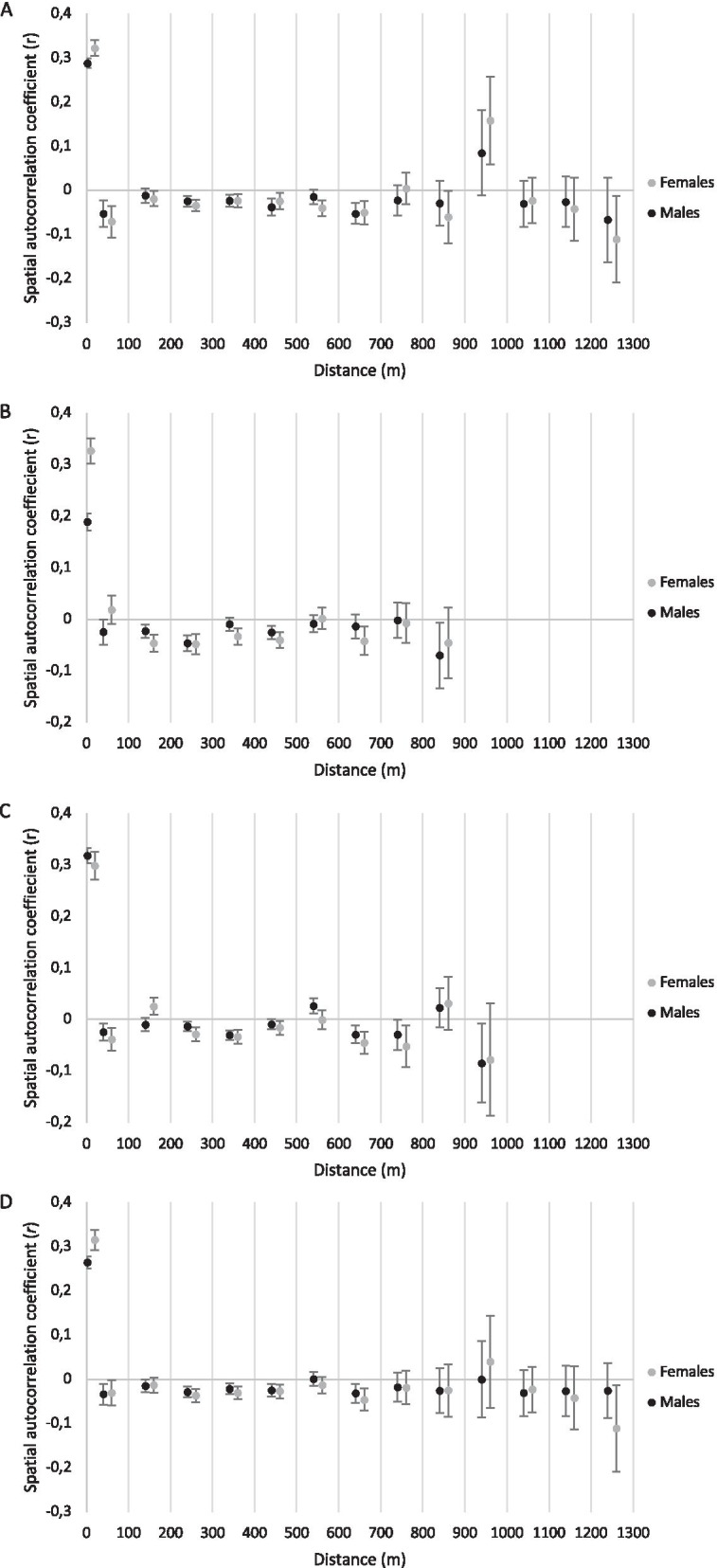
Spatial autocorrelation for the 399 individuals from 47 colonies analysed in this study, including 193 individuals from 23 colonies in 2004 (**A**), 146 individuals from 27 colonies in 2005 (**B**), 208 individuals form 34 colonies in 2006 (**C**), and all three datasets (2004, 2005, 2006) combined (**D**). A total of 13 distance classes of 100 m increments were specified, since the maximum distance between any two colonies in our study site was 1227 m. Intra-colony estimates are indicated at 0 m distance. Points represent mean spatial autocorrelation coefficients for each distance class, for males and females, and error bars represent 95% confidence intervals. Error bars that do not overlap zero represent significant genetic structure. Dotted lines represent linear trendlines for males (black) and females (grey). **D** The slope of regression (change in *r* per km) is steeper overall for females (− 0.1) than for males (− 0.08)

Spatial autocorrelation analysis revealed significant positive genetic structure within colonies, among both males and females (Fig. 4; see 0 m distance in all three datasets, A–C, and overall, D), indicating a higher level of relatedness within colonies than would be expected under random mating, which is expected in cooperatively breeding species. Across all 3 sampling years, the mean intra-colony relationship coefficient, *r*, for the 47 colonies, calculated using 399 individuals, was high (0.290; Table 2). In contrast, inter-colony *r* was negative (− 0.022; Table 2), indicating a low level of relatedness among colonies, also in line with expectations for this species.

Intra-colony *r* was significantly higher among females than males in 2004 (females: 0.322; males: 0.287;* p* = 0.023; Table 2) and in 2005 (females: 0.327; males: 0.189;* p* = 0.002; Table 2), and did not differ significantly between sexes in 2006. On average, across all 3 years, intra-colony *r* was significantly higher among females than males (females: 0.315; males: 0.264;* p* = 0.022; Table 2). In all 3 years, male–female intra-colony *r* values were between those of males and females (2004: *r* = 0.305; 2005: *r* = 0.248; 2006: *r* = 0.292). Inter-colony* r* was slightly higher among males than females in each year, and significantly higher on average across all 3 years (males: − 0.020; females: − 0.026; *p* = 0.011; Table 2).

### Isolation by distance

In all 3 years, intra-colony *r* was high (mean 0.2899) and inter-colony *r* low (mean − 0.022), irrespective of distance between colonies (Table 2, Fig. 4). Linear regression, i.e. slope of the relationship between spatial distance and genetic relatedness, across all distance classes (excluding intra-colony) was low (not significantly different from zero; Table 2) for all datasets, hence we did not find evidence for IBD. However, it is worth noting that negative regression (*blog*) was consistently more pronounced among females than males (Table 2; Fig. 4).

### Dispersal estimates from genetic data

Effective population density, *D*, was estimated for each dataset based on the number of individuals captured in the 0.85 km^2^ (1700 m × 500 m) study site. Neighbourhood size (*Nb*) and sigma (σ) estimates were obtained for the 2004 and 2006 male datasets, and the dataset comprising all males from all 3 years, while estimates for the female datasets and the 2005 male dataset did not converge, and therefore could not be estimated (Table 2). Sigma (σ) estimates of 0.135, 0.207 and 0.135 for the 2004, 2006 and combined male datasets respectively translated to dispersal distance estimates of 190 m, 293 m and 191 m respectively.

### Effects of ecological constraints on dispersal and colony fission

Average annual rainfall in Tswalu for the 6 years preceding the current study period (1998–2003) was 300.5 mm ± 110.8 mm. Annual rainfall for 2004, 2005 and 2006 was 247.9 mm, 422.7 mm and 532.6 mm respectively. Thus, rainfall in 2004 was below average, 2005 above average, and 2006 substantially above average.

Our mark-recapture data revealed 36 dispersal events across the 3 sampling years, of which two were detected within the 2004 sampling period, 11 between 2004 and 2005, 21 between 2005 and 2006, and a further two dispersals some time between 2004 and 2006 (these individuals were not captured in 2005, and therefore it is impossible to say exactly when they dispersed; Fig. 1; Additional file 1: Table S1). Since we do not have dispersal data preceding 2004, we could only compare observed dispersal from 2004 to 2005 with that of 2005 to 2006, and this test revealed a significant increase in the number of dispersal events between these sampling periods (*P* = 0.016).

**Fig. 5:**
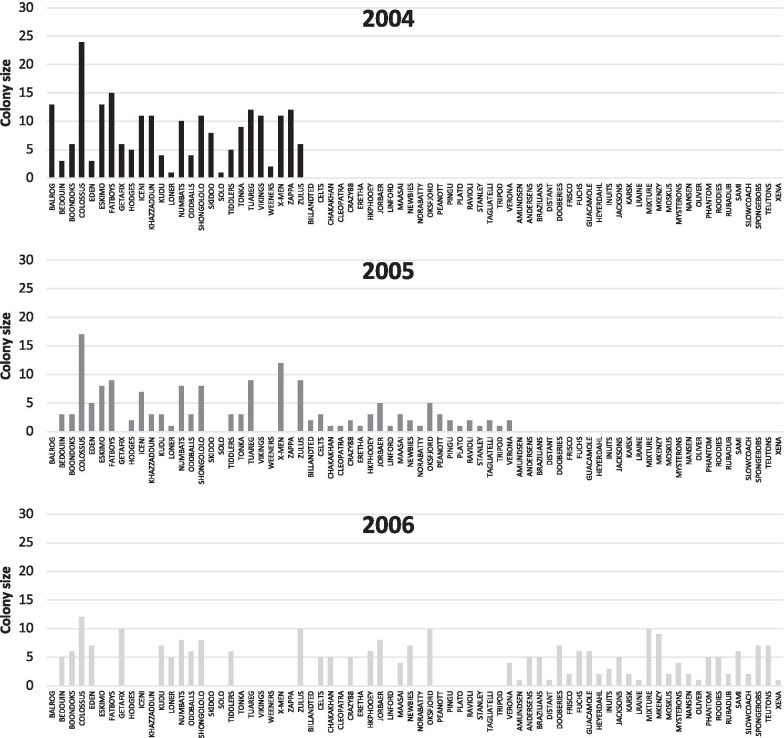
Colony representation across the three sampling years 2004, 2005 and 2006. Colonies Balrog through Zulus were represented in 2004, with new colonies Billandted through Verona arising in 2005, and further new colonies Amundsen through Xena arising in 2006. Of the 26 colonies sampled in 2004, 19 persisted in 2005, and 12 of these still persisted in 2006. Of the 21 new colonies arising in 2005, only eight persisted in 2006, with a further 28 new colonies arising that year

**Fig. 6:**
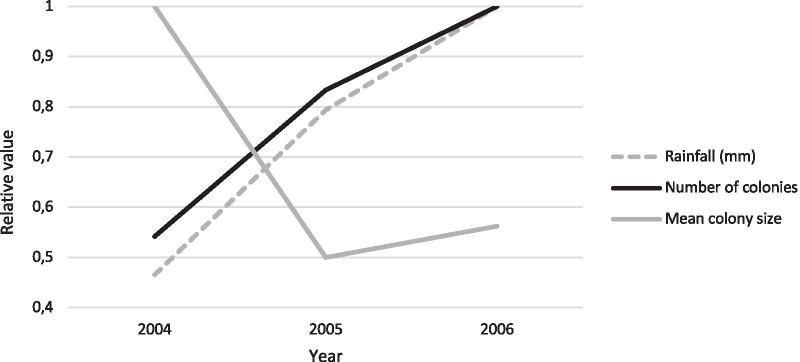
Colony fission in response to rainfall. Y-axis values are shown as relative values, for each of three measurements (rainfall, number of colonies and mean colony size), i.e. as a proportion of the highest value in each given measurement category, e.g. in 2005 mean colony size was half (0.5) that of 2004. Colony numbers increase and sizes decrease with increasing annual rainfall between 2004 and 2006, indicative of colony fission along with subsequent colony growth through reproduction

In our study population, 2004 was associated with relatively few colonies of large sizes, while 2005 and 2006 were characterized by successively larger numbers of smaller colonies, comprising many newly established colonies (Fig. 5). In 2004, a relatively small number of colonies [26] of relatively large size (mean = eight) existed, most of which persisted in 2005 (19/26 = 73%), along with the addition of a few [21] newly established colonies (total = 40), some arising from colony fission (Figs. 5 and 6). Average colony size in 2005 dropped from eight to four. In 2006, we observed further colony fission, with large established colonies decreasing in size as a result of emigration of colony members, colonies founded in 2005 increasing in size due to reproduction (in total 20 established colonies persisted, of which 12 had persisted since 2004), and yet more new colonies being established and already at a fair size due to reproduction. The average colony size thus rose again to 4.5 in 2006, although with many [28] newly established colonies (total = 48), resulting in a substantial increase in overall population size. Thus, we also observed a significant increase in the number of newly established colonies, i.e. colony fission events (*P* = 0.001).

## Discussion

### Spatial genetic structure and local dispersal

Both population-level *F*_ST_ analysis and individual-level spatial autocorrelation analysis revealed strong signals of population genetic structure, with high levels of relatedness within colonies. This is not unexpected in a cooperatively breeding species, and is commonly associated with natal philopatry in social vertebrates, in which offspring of one or both sexes frequently inherit the breeding position in their natal group [51–56].

We did not find evidence for IBD at the spatial scale investigated here. Spatial autocorrelation of relatedness revealed that relatedness among individuals from colonies in close proximity, less than 200 m apart is not substantially higher than that of individuals from colonies at intermediate distances up to 800 m apart, but is instead relatively constant (close to zero) across this spatial range, and only begins to decrease at distances greater than 800 m (Fig. 4). Thus, it seems plausible that gene flow is well maintained at distances of up to 800 m, and that IBD may become significant at greater distances, however this hypothesis is yet to be tested on a dataset representing a larger geographic range.

Observational data revealed a mean dispersal distance of 177 m ± 150 m, which is well below the mean distance between colonies in our site (363 m ± 210 m), indicating that both males and females typically disperse to nearby colonies, rather than undertaking long distance dispersal. However, since this finding is not explicitly supported by the genetic data, the frequency of long-distance dispersals may be underestimated, particularly given the possibility that some individuals may disperse out of the study area. In our study few individuals undertook long-distance dispersals greater than 400 m (sample #64: 456 m; #461: 472 m; #204: 530 m; Additional file 1: Table S1, Fig. 1). Since these and other dispersals cross the territories of multiple other established colonies, it is likely that these may represent aboveground dispersal events. Finn [57] hypothesized that dispersal distances greater than 250 m are likely to represent aboveground dispersal in Damaraland mole-rats, due to the high energetic costs associated with digging. Long-distance dispersal can thus be risky, exposing individuals to predation, aggressive interactions with conspecifics, loss of body condition and stress [31, 39, 58], while also trading off against cooperative contributions that they might otherwise have made within their natal group [59]. Local dispersal may also involve fitness costs, arising from kin competition [60] and/or exposure to a risk of inbreeding [61, 62]. On the other hand, local dispersal could be facilitated by familiarity with individuals in the destination group [63], while colony fission, leading to the establishment of new breeding colonies in close proximity to the natal colony could be facilitated if relatives within the natal group were more accepting than potentially aggressive and territorial non-relatives elsewhere [61, 64]. The high frequency of dispersals between colonies Hodges, Tuareg and Mkenzy could be a reflection of this type of local dispersal between neighbouring colonies, with the establishment of the new colony, Mkenzy, in 2006 (Fig. 1). More specifically, the movement of a male and female (#113 and #204; Fig. 1, Additional file 1: Table S1) from Tuareg to Hodges, and the converse (#399 and #400; Fig. 1, Additional file 1: Table S1), could indicate that individuals (possibly potential breeding pairs) are dispersing together. Torrents-ticó et al*.* [37] found that 121 (52 female, 69 male) out of 153 *Fukomys damarensis* dispersers (79%) dispersed to establish new colonies, and that 28 (7 female, 21 male; 18%) dispersed in coalition (along with other individuals of the same sex).

### Male-biased dispersal

Both our observational data and genetic data are in line with male-biased dispersal in this species. We did not detect a skew in the number of male vs female dispersers, but present multiple lines of evidence that males typically disperse farther than females and are more likely than females to disperse to other established colonies, as opposed to founding new colonies. The observational data revealed that males dispersed on average 215 m ± 180 m, while females typically dispersed only 139 m ± 104 m, although this difference is non-significant. The genetic data were not powerful enough to accurately estimate σ for females in any given year, however, we were able to estimate σ for males in 2004, 2006 and across all 3 years. The mean predicted dispersal distance across all 3 years as inferred from the genetic data (191 m) is slightly lower than the mean estimate obtained from observational data for males (215 m ± 180 m), but still substantially higher than the mean estimate for females (139 m ± 104 m). Interestingly, of the 36 (19 male; 17 female) dispersal events, only four represented distances greater than 400 m, and all of these were males. Female dispersals were typically associated with establishment of new colonies (e.g. colony fission events), with only 12% of dispersal events involving immigration into neighbouring colonies, whereas males were found to disperse more frequently into established colonies (32% of male dispersal events). Another study has similarly demonstrated a high proportion of immigrants within established colonies of *F. damarensis* (4.3% of nonbreeding colony members at Dordabis and 13.9% at Hotazel) and *F. anselli* (3.5% at Lusaka), although this study did not specifically analyse the proportion of male vs. female immigrants.

Population-level *F*_ST_ analysis revealed a higher mean *F*_ST_ among females than males, indicating significantly greater genetic differentiation among females than males (*p* < 0.001), a pattern consistent with females being the more philopatric sex, and males being more dispersive. In 2004 and 2006, the difference between male and female *F*_ST_ became more pronounced with increasing spatial distance, consistent with the idea that males disperse over greater distances than females. Furthermore, spatial autocorrelation analysis indicated that on average, across all 3 years, intra-colony relatedness was significantly higher among females than males, while inter-colony relatedness was significantly lower. This is consistent with diminishing spatial genetic structure among males caused by long-distance male dispersal [44]. IBD was consistently more pronounced among females than males, consistent with the hypothesis that males are the more dispersive sex, and may disperse over greater distances than females.

The asymmetry of dispersal between the sexes observed in this study is somewhat consistent with the results of previous studies in social mole-rats. Genetic studies of the colony compositions of social mole-rats have shown that unrelated male non-breeders may be present in the colonies, suggesting that males may occasionally immigrate into colonies in the wild [65–67]. A study of Damaraland mole-rats that drowned in a channel suggested that males may more readily disperse than females [36]. Furthermore, a recent study of this species demonstrated that males disperse earlier (males: 371 days ± 257; females: 411 days ± 279) and more frequently (males: 96 individuals; females: 60 individuals) than females [37]. However, a recent study of Damaraland mole-rats from the Kalahari (including a population at the Kalahari Research Centre and the current population at Tswalu), found a higher number of females (31 out of 41 female dispersers, i.e. 76%) taking on long-distance dispersal (> 250 m) than males (27 out of 47 male dispersers, i.e. 57%), although the number of male dispersers (47 individuals) was still higher overall than females (41 individuals) [57].

Several other studies of social mole-rat species have demonstrated that males may be more likely to disperse and may disperse further, whereas females may be more likely to undertake local dispersal to establish a territory in close proximity to their natal group [67–71].

### Effects of ecological constraints on dispersal and colony fission

The compacted-sand substrate characterising the arid ecoregions inhabited by Damaraland mole-rats is extremely costly to work [72] and poses an ecological constraint that is thought to have favoured the evolution of delayed dispersal and cooperative breeding [25, 27, 29]. However, during periods of sustained rainfall, this constraint relaxes significantly, and the softened sands stimulate increased dispersal by subordinates of both sexes (either to immigrate to other existing colonies or to establish new colonies; [25, 36, 65]). Physiological suppression among subordinate females eases during the annual rains, when ecological constraints on dispersal are relaxed, despite the continued presence of the dominant female and in groups that contain no new immigrant males [40]. This is not only because the subordinate female’s own chances of successful dispersal are higher, but because her chances of encountering mating opportunities with unrelated males are also increased [25, 36, 65].

Our results demonstrate elevated dispersal and colony fission associated with increased rainfall. Although the mark-recapture data revealed only 36 “dispersal events”, of which eight represented dispersals into established colonies, and 28 represented colony fissions, we observed a total of 49 newly established colonies across the 3-year sampling period, indicating that colony fission was greater than the mark-recapture data revealed. This is because “dispersal events” were only recorded if individuals were captured multiple times in different colonies, and some individuals may have evaded recapture, or been involved in colony fissions without having been previously captured in their natal colony. Colony fission in this population would involve individuals moving out of larger established colonies, in which they were most likely reproductively suppressed and involved in altruistic co-operative activities, to establish new colonies by means of colony genesis (pairing with an unrelated mate and establishing a new family group; [26]), thereby increasing the number of reproductively active animals in the overall population and consequently reducing reproductive skew. This finding supports the hypothesis [27] that environmental factors, such as rainfall, may play a significant role in the maintenance and/or disruption of reproductive skew in this species.

The structure of social groups in species with dispersal asymmetry can be described by two important parameters, i.e. the mean number and the mean size of the groups [1], although when considering hypotheses for the evolution of sociality, it is important to note that these life-history parameters depend directly upon environmental conditions. Resource availability (food, water, shelters, space), as well as abiotic conditions (climate) and biotic environment (density of predators, allospecific competition), directly influence birth and survival rates [1, 73]. In social species with dispersal asymmetry and female philopatry, colony fission (e.g. involving a single male and female “budding” from their native colony into a neighbouring territory as described in our study, as opposed to long-distance dispersal occasionally undertaken predominantly by males), may be seen as a way for females to disperse. Through colony fission, females can leave the other females in their natal colony, their own relatives, and even their natal home range [1]. Moreover, the critical size for fission to occur is seldom reached under unfavourable demographic and environmental conditions (often characterising arid habitats), thus further driving delayed dispersal, colony growth and cooperative breeding and/or reproductive skew in these species.

## Methods

### Study site and sampling

*Fukomys damarensis* were live trapped at Tswalu Nature Reserve in the southern Kalahari (27° 12.855′ S 22° 27.364′ E), South Africa using Hickman tunnel traps [74]. The 1700 m × 500 m study site (Fig. 1) comprised a total of 74 temporally variable burrow systems, or colonies, which were sampled biannually (both in the wet and dry season) over a period of 3 consecutive years (2004–2006).

Active burrows were located by excavating one or two of the surface mounds that essentially radiate out from the centre of a colony like the spokes of a wheel. Excavated holes were left open with wire mesh inserted to prevent the entry of mole snakes and if further activity was observed in the form of animals blocking the excavated holes from the inside, traps were set after removal of the soil and mesh. Each colony was usually trapped from a single active hole, however, in some cases multiple holes in close proximity were found to belong to a single colony, which was determined by the presence of a single breeding pair. Breeding females were easily recognised by a perforate vagina and swollen teats, and breeding males by a stained mouth and bulging inguinal testes (24, as validated by 65), and the sexes were differentiated by the shape of their genitalia [24].

By continually running trap sites for multiple days and keeping trapped individuals in captivity (in plastic bins lined with fresh sand and provisioned with fresh sweet potato), entire colonies were trapped out. Colonies were considered “trapped out” when no further activity was noted for 2 consecutive days. Animals were marked for recapture in subsequent sampling periods using a unique toe-clipping system for identification. At the time of the study microchips and readers were not available for marking animals. Sex, mass, colony membership and GPS co-ordinates were recorded for each capture, and skin biopsies were stored for genetic analysis. After sampling, all individuals were returned to their burrow systems.

A total of 789 captures were made over the 3-year period, representing 486 unique individuals (excluding re-captures). Trapping sites differed between sampling periods, but individual identification of breeding females through mark-recapture facilitated identification of colonies, even if the burrow system had spatially shifted slightly between sampling periods, or if the sampling site represented a different portion of the burrow system. Hence GPS co-ordinates, representing the actual trap site, of some colonies differed slightly from 1 year to the next.

### Genotyping

Whole genomic DNA was extracted from the collected tissue samples using the phenol/chloroform extraction method [75]. PCR amplification was carried out in 96-well plates (in total volumes of 10 µL) as follows: 0.5 U of Super-Therm® DNA polymerase (Southern Cross Biotechnology), 1× Buffer (Southern Cross Biotechnology), 0.2 mM of each of the four dNTP’s (Promega) 1.5 pM of each primer, approximately 20 ng of DNA and 0.6–1.5 mM MgCl_2_ per reaction. Genotypes were generated on an ABI automated sequencer (Applied Biosystems) and analysed using GENEMAPPER Version 3.0 (Applied Biosystems).

Genotypic profiles were generated for all 486 captured individuals using seven genus-specific microsatellite loci (Table 1) after testing 11 loci developed by Burland et al*.* [76]. Of the 11 markers tested, two were omitted from subsequent analyses due to high levels of null alleles (CH1 and CH4), one due to poor amplification success in our study (CH2), and one due to potential sex-linkage (DMR1). Modifications were made to three of the primer pairs (DMR1, DMR3 and DMR7) to facilitate co-loading on the ABI 3100 sequencer during GENESCAN® analyses. The loci were renamed to DMRN1, DMRN3 and DMRN7, respectively. Primers were 5′ fluorescent labelled (The Scientific Group). Allele frequencies, observed and expected heterozygosity were calculated for each marker using Cervus (Version 3.0.7), and assessed for Hardy–Weinberg equilibrium and the presence of null alleles.

**Table 1:** Marker data for 7 microsatellite markers (Burland et al*.* [76]) employed in this study

Marker	Dye	Size range	T_A_	% successfully scored	*k*	*H*_O_	*H*_E_	HW	*F* (null)
CH3	PET	120–136	54.6	82.7	13	0.864	0.872	NS	0.0046
DMR2	6-FAM	151–169	61.7	80.8	13	0.870	0.854	NS	− 0.0092
DMRN3^a^	VIC	126–154	57.6	90.3	13	0.841	0.869	***	0.0183
DMR4	NED	205–225	57–55	83.2	19	0.796	0.825	***	0.0210
DMR5	6-FAM	244–274	57–55	78.3	18	0.809	0.777	*	− 0.0222
DMR6	NED	123–147	57–55	87.2	10	0.465	0.866	***	0.3040
DMRN7^a^	VIC	132–148	60.3	83.2	9	0.826	0.834	*	0.0058

*k*: number of alleles in 486 genotyped individuals; *H*_O_: observed heterozygosity; *H*_E_: expected heterozygosity; HW: Hardy–Weinberg equilibrium; NS: not significant; F (null): frequency of null alleles

^a^Newly designed F primers

### Dispersal estimates from mark-recapture data

Mark-recapture data were collected throughout the study period (2004–2006), and dispersal events were recorded when the same individual was captured in more than one colony, either in the same sampling year, or in successive years. In some cases, dispersals within a sampling period were verified by recapture of that individual in the new colony in a subsequent sampling period, but this was not true for all dispersals, therefore we do not rule out the possibility that some of these recorded “dispersals” do not represent true dispersal events, but rather other movements or forays (such as unsuccessful breeding attempts, or simply foraging forays) [77], between colonies. However, since this study was conducted alongside a genetic investigation into gene flow, essentially reflecting only reproductively successful dispersal events, we considered all recorded movements of individuals between colonies as relevant observational data. We also acknowledge that some dispersing individuals may have evaded recapture, and that our data thus represent only a subset of the total pool of dispersers within the population.

Body mass was used as an indicator of age; individuals weighing more than 80 g were deemed “adults”, between 50 and 80 g “sub-adults”, and less than 50 g “juveniles”. These categories were used to distinguish natal dispersal from other dispersal events. GPS co-ordinates of capture sites were used to compute Euclidean dispersal distances. We assessed the significance of sex differences in mean dispersal distance, and frequency of dispersal across twelve distance classes of 50 m increments (0–600 m) using paired two-tailed t-tests, with the significance level set to 0.05.

### Spatial genetic analysis

Mark-recapture data was verified using genotypic data from seven microsatellite loci (Table 1). All 486 captured individuals were genotyped, but only the 399 individuals that passed the maximum missingness threshold of 50% were analysed. Thus, the 3 sampling years were analysed separately, with 193 individuals representing 23 colonies for 2004, 146 individuals representing 27 colonies for 2005 and 208 individuals representing 34 colonies for 2006 (Table 2). GPS co-ordinates were used to compute distances between colonies and between individuals, and 13 distance classes of 100 m increments were specified, with the first class representing distances up to 100 m, and the final class representing distances between 1200 and 1300 m (the maximum distance between any two colonies in our study site was 1227 m). Pairwise *F*_ST_ [78] was estimated using a nested ANOVA [79] to assess spatial autocorrelation of genetic variance among colonies. In the case of cooperative breeders, *F*_ST_ represents the proportion of genetic variance that is partitioned among different social groups, or colonies. Low *F*_ST_ values imply that colonies are genetically similar, whereas high values suggest that colonies are genetically more distinct, and the lower the rate of gene flow between colonies, the higher the *F*_ST_ value. Since *F*_ST_ values depend not only on the amount of differentiation among populations, but also on the diversity within, which may be influenced by the choice of marker, there is no “rule” as to what constitutes high or low *F*_ST_. However, in broad terms *F*_ST_ < 0.05 may be considered as generally low genetic differentiation, 0.05–0.15 as moderate, 0.15–0.25 as great and > 0.25 as very great, thus *F*_ST_ > 0.15 may generally be considered as significant differentiation [80, 81]. Sex-biased dispersal can be assessed by calculating *F*_ST_ separately for males and females, and the more philopatric sex is expected to show higher *F*_ST_ values [44]. Significance of differences in *F*_ST_ between colonies represented by males and females across all distance classes was assessed by permutation analysis as implemented in SPAGeDi v.1.5 (Spatial Pattern Analysis of Genetic Diversity; [82]), and paired t-tests.

**Table 2:** Pairwise *F*_ST_, spatial autocorrelation coefficients (*r*) and IBD analysis for males and females from each sampling year (2004–2006)

Analysis	*N* colonies	*N* individuals	*F*_ST_	DF	*P*	Intra-colony *R*	DF	*P*	Inter-colony *R*	DF	*P*	IBD analysis			
												*Rxy*	*P*	*blog*	*P*
2004 females	23	80	0.172	228	0.108	0.3219	132	0.023*	− 0.030	3021	0.090	− 0.026	0.459	− 0.007	0.537
2004 males	23	113	0.185			0.2874			− 0.026			− 0.032	0.320	− 0.006	0.572
2005 females	27	65	0.162	306	< 0.001*	0.3265	78	0.002*	− 0.032	1937	0.206	0.012	0.281	− 0.006	0.541
2005 males	27	81	0.117			0.1886			− 0.022			0.013	0.269	0.009	0.309
2006 females	33	83	0.185	501	< 0.001*	0.2978	77	0.342	− 0.020	3269	0.538	− 0.033	0.223	− 0.009	0.321
2006 males	34	125	0.161			0.3173			− 0.014			− 0.011	0.452	0.003	0.716
All females	47	163	0.175	1035	< 0.001*	0.3154	295	0.022*	− 0.026	8229	0.011*	–	–	–	–
All males	47	236	0.153			0.2644			− 0.020			–	–	–	–
Males and females	47	399	0.167	–	–	0.2899	–	–	− 0.022	–	–	–	–	–	–

*N* colonies: number of colonies in each analysis. i.e., those containing at least two same-sex individuals for single-sex analyses; *N* individuals: total number of individuals in each analysis; *F*_ST_: the estimated amount of genetic variation among colonies; intra-colony *r*: the estimated level of relatedness within colonies; inter-colony *r*: the estimated level of relatedness among colonies. Significant differences between males and females are indicated by an asterisk. along with associated *p* value (alpha = 0.05) and degrees of freedom (df). IBD analyses for each of the six datasets are shown as regression of pairwise *r* on the logarithm of spatial distance (*Rxy*, as computed in Genalex, and *blog*, as computed in Spagedi) with the associated p-values

In addition to population-level *F*_ST_, pairwise relatedness coefficients (*r*; [83]) were used to analyse fine-scale spatial genetic structure by assessing spatial autocorrelation of relatedness among individuals. Significance of differences in relatedness among males vs. females across all distance classes (including within colonies) was once again tested by permutation analysis as implemented in SPAGeDi [82], and paired t-tests.

### Isolation by distance

We used both SPAGeDi v.1.5 [82] and GenAlEx v.6.5 [84] to test isolation by distance (IBD) separately in males and females for each of the 3 sampling years. Pairwise relatedness coefficients (*r*) and spatial distance matrices, as computed in SPAGeDi, were used to perform Mantel tests in genalex. Significance was assessed using 999 random permutations in genalex, and 1000 permutations in SPAGeDi.

Theoretical models of IBD show that, under certain conditions, relatedness coefficients between individuals are expected to vary approximately linearly with the logarithm of the distance in a two-dimensional space, and with the linear distance in a one-dimensional space [85, 86]. Thus, a negative regression, i.e. slope of the relationship between spatial distance and genetic relatedness reflects increasing IBD, and is expected to be steeper in the more philopatric sex, for which relatedness decreases more rapidly with increasing spatial distance.

### Dispersal estimates from genetic data

The gene dispersal distance parameter, sigma (σ), was estimated from the regression of pairwise relationship coefficients (or Rousset distance) on the logarithmic distance, with the assumption that genotypes come from a two-dimensional population at drift-dispersal equilibrium such that theoretical expectations of isolation-by-distance models hold [82, 85, 86]. SPAGeDi uses an iterative procedure to determine σ (the square root of half the mean square parent–offspring distance) and *Nb* (neighbourhood size) by regressing pairwise relationship coefficients on ln(distance) over a restricted distance range. The procedure requires an estimate of the effective population density, *D*, as well as *X*, the width of the distance range σ to *X*σ over which the regression is applied. Starting from a global regression slope (*blog*), the procedure consists in estimating *Nb* as *Nb* = − (1 − *F*_(1)_)/*blog*, where *F*_(1)_ is the relationship coefficient between individuals for the first distance class (assumed to correspond to pairs of neighbours; the intra-colony class is excluded), and σ is estimated as σ = [*Nb*/(2*π*·*k*·*D*)]^1/2^. Then, restricting the regression (*blog*) to distances between σ and Xσ, *Nb* and σ are estimated again. This step is repeated until σ converges, with up to 100 iterations [82]. Convergence is not ensured, in which case no estimate is provided.

### Rainfall data collection

Rainfall data for Tswalu Kalahari Reserve were provided by the Tswalu Foundation. Dispersal and spatial genetic data were evaluated in light of the fluctuation in annual precipitation averages. Chi-squared tests were used to test the statistical significance of differences in frequency of dispersal and colony fission events between sampling periods (2004–2005 and 2005–2006), and thus the association of these parameters with annual rainfall.

## Supplementary Information

**Additional file 1: Table S1.** Evidence of dispersal from mark-recapture data, indicating the colonies/capture sites for individuals that were captured at different sites in successive years, and the spatial distance associated with each dispersal event.

## Data Availability

The dataset supporting the conclusions of this article is available in Dryad [Microsatellite genotypes for Damaraland mole-rats (*Fukomys damarensis*) from the southern Kalahari] at 10.5061/dryad.8sf7m0ckq.
