# Supplementary material for: Spatial population genetic structure and colony dynamics in Damaraland mole-rats (Fukomys damarensis) from the southern Kalahari
Source: BMC Ecol Evol. 2021 Dec 8;21:221. doi: 10.1186/s12862-021-01950-2 (PMC8653584; doi:10.1186/s12862-021-01950-2)
Supplement: Supplementary file 1 — Additional file 1: Table S1. Evidence of dispersal from mark-recapture data, indicating the colonies/capture sites for individuals that were captured at different sites in successive years, and the spatial distance associated with each dispersal event. [file 12862_2021_1950_MOESM1_ESM.docx]

**Table S1**: Evidence of dispersal from mark-recapture data, indicating the colonies / capture sites for individuals that were captured at different sites in successive years, and the spatial distance associated with each dispersal event.

| Sample no | Body mass | Sex | 2004 | 2005 | 2006 | Distance (m) | New / established colony |
| --- | --- | --- | --- | --- | --- | --- | --- |
| 29 | 125 | F | ESK | ESK/LIN | - | 393 | N |
| 65 | 96 | F | ODD | ODD/PEA | - | 228 | N |
| 115 | 98 | F | TON | - | TEU | 198 | N |
| 122 | 95 | F | TUA | TUA | MKE | 107 | N |
| 131 | 94 | F | TUA | TUA | MKE | 107 | N |
| 178 | 123 | F | FAT | CHA | CHA | 163 | N |
| 182 | 69 | F | FAT | FAT | XEN | 145 | N |
| 184 | 117 | F | FAT | FAT/ERE | - | 342 | N |
| 194 | 38-134 | F | KHA | - | GUA | 37 | N |
| 197 | 91 | F | XME | XME | AND | 106 | N |
| 202 | 84 | F | XME | XME | PHA | 146 | N |
| 342 | 58 | F | - | ESK | INU | 60 | N |
| 399 | 66 | F | - | HOD/TUA | - | 21 | E |
| 402 | 74 | F | - | TUA/HOD | - | 21 | E |
| 421 | 36 | F | - | ICE | RUB | 122 | N |
| 490 | 103 | F | SKI | PIN | - |  | N |
| 667 | 72 | F | - | - | MIX/PHA | 53 | N |
| 46 | 68 | M | COL | COL | PHA | 245 | N |
| 64 | 135 | M | COL/ZAP | COL/XME | AND | 456, 149, 143 | N?, E, N |
| 69 | 162 | M | COL | COL/XME | COL | 149 | E, E |
| 105 | 86 | M | ICE/WEE | - | - | 145 | N |
| 112 | 108 | M | HOD | HOD | MKE | 107 | N |
| 113 | 141 | M | TUA | TUA/HOD | - | 21 | E |
| 169 | 121 | M | FAT | XME | - | 111 | E |
| 175 | 167 | M | FAT | FAT | MIX | 236 | N |
| 204 | 74 | M | XME | XME | LON | 530 | N |
| 249 | ? | M | NUM | NUM | TEU | 198 | N |
| 400 | 48 | M | - | HOD/TUA | MKE | 21, 107 | E, N |
| 423 | 154 | M | - | BIL | RUB | 151 | N |
| 461 | 80 | M | - | SHO | HEY | 472 | N |
| 475 | 104 | M | - | STA | MIX | 48 | N |
| 493 | 141 | M | - | PEA | GUA | 22 | N |
| 644 | 101 | M | - | - | MIX/PHA | 53 | N |
